# A novel citrate-based protocol versus heparin anticoagulation for sustained low-efficiency dialysis in the ICU: safety, efficacy, and cost

**DOI:** 10.1186/s12882-018-0879-4

**Published:** 2018-04-03

**Authors:** Ming Wen, Claudius Küchle, Dominik Steubl, Robin Satanovskji, Uwe Heemann, Yana Suttmann, Susanne Angermann, Stephan Kemmner, Lisa Rehbehn, Monika Huber, Christine Hauser, Christoph Schmaderer, Anna-Lena Reichelt, Bernhard Haller, Lutz Renders

**Affiliations:** 1 Department of Nephrology, Klinikum rechts der Isar, Technische Universität München, Munich, Germany; 2 Institute of Medical Statistics and Epidemiology, Technische Universität München, Munich, Germany

**Keywords:** Regional citrate anticoagulation, Sustained low-efficiency dialysis, Critically ill patients

## Abstract

**Background:**

The high cost, complexity of the available protocols, and metabolic complications are the major barriers that impede the clinical utilization of regional citrate anticoagulation (RCA) for sustained low efficiency dialysis (SLED) in critically ill patients. By comparing a novel protocol for SLED using 30% citrate solution with common protocol using unfractionated heparin, this study aimed to provide new insights for clinical applications of RCA.

**Methods:**

In this retrospective study, a total of 282 critically ill patients who underwent SLED with citrate and/or heparin anticoagulation in six adult ICUs were enrolled. These patients were divided into three groups based on the anticoagulation regimens they had received during the treatment in ICU: Group 1 (Citrate) had only received treatment with citrate anticoagulation (*n*=75); Group 2 (Heparin) only with heparin anticoagulation (*n*=79); and Group 3 (Both) with both citrate and heparin anticoagulation (*n*=128). We compared the mortality, metabolic complications as well as cost among these groups using different anticoagulation regimens.

**Results:**

The in-hospital mortality did not significantly differ among groups (*p*> 0.1). However, three patients in heparin group suffered from severe bleeding which led to death, while none in citrate group.

Overall, 976 SLED sessions with heparin anticoagulation and 808 with citrate were analyzed. The incidence of extracorporeal circuit clotting was significantly less in citrate (5%), as compared to that in heparin (10%) (*p*< 0.001). Metabolic complications and hypotension which led to interruption of SLED occurred more frequently, though not significantly, in citrate (*p*= 0.06, *p*= 0.23).

Furthermore, with 30% citrate solution, the cost of anticoagulant was reduced by 70% in comparison to previously reported protocol using Acid Citrate Dextrose solution A (ACD-A).

**Conclusions:**

Our results indicated that anticoagulation regimens for SLED did not significantly affect the mortality of patients. Citrate anticoagulation was superior to heparin in preventing severe bleeding and circuit clotting. The protocol adopted in this study using 30% citrate solution was safe as well as efficacious. In the meantime, it was much more cost-efficient than other citrate-based protocol.

## Background

As a result of technical advancements over the last decade, sustained low-efficiency dialysis (SLED) has become an effective and safe treatment for critically ill patients in intensive care units (ICUs). Due to its simplicity, convenience and low cost, SLED is widely adopted [1–3]. However, a high incidence of circuit clotting was reported in 26 to 46% of treatments without anticoagulation. Using unfractionated heparin, the circuit clotting rate could be significantly reduced to 17–26% [2, 4–7]. Thus, anticoagulation is essential for preventing the extracorporeal circuit clotting in SLED.

The standard regimen with unfractionated heparin has been well established but associated with an increased risk of bleeding [8, 9]. Against this background, regional citrate anticoagulation (RCA) was advocated as an ideal alternative to systemic heparin anticoagulation for patients at risk of bleeding. The advantages of citrate anticoagulation, including longer circuit survival, reduced bleeding risk, and possible improvement of patient mortality, have been reported in continuous renal replacement therapy (CRRT) [9–13]. Therefore, the 2012 Kidney Disease Improving Global Outcomes (KDIGO) Clinical Practice Guidelines recommend the use of RCA as the preferred anticoagulation modality in critically ill patients [14].

Though RCA for SLED has been reported to be a safer alternative to heparin, several issues remain. First of all, previous studies regarding citrate anticoagulant for SLED were either small, focused on a subset of patients in the ICU (patients with severe burn injuries, patients without risk of bleeding), or lacked a control group [10, 15–17]. Secondly, only three studies described their protocols, using acid citrate dextrose solution A (ACD-A, Fresenius: 3% citrate, 0.8% citric acid, 2.2% trisodium citrate, 112.9 mmol/l total citrate anion in 2.5% dextrose) or 4% sodium citrate solution [10, 17, 18]. Furthermore, those protocols could result in additional fluid load. Ponikvar et al. reported a significant reduction of infused volume during plasma exchange using 15% citrate solution as compared to 4% citrate solution [19]. Although unproven, increased solute and fluid shifts are commonly considered as a risk factor for intradialytic hypotension [20]. Last but not least, the aforementioned protocols are expensive. The costs of SLED with citrate anticoagulation are assumed to be higher than with heparin, which also precludes their implementation into clinical routine.

Given the above mentioned concerns with RCA, physicians are still cautious in its implementation.

## Methods

In this study, we describe a novel citrate-based protocol for SLED using 30% citrate solution which has been performed since 2010 in our hospital. Safety and efficacy of this protocol were compared with systemic heparin anticoagulation. Additionally, we made a direct comparison of the cost of various protocols, which has not been tackled in previous studies.

### Patients and clinical data

We retrospectively analyzed data from all critically ill patients who underwent SLED at six adult ICUs (general, surgical, neurological) in our university hospital between January 2013 and August 2015. Patients were excluded if a heparin- induced thrombocytopenia was diagnosed or SLED was performed with other anticoagulants, without any anticoagulation, or longer than 12 h.

To assess the mortality, patients were grouped according to dialysis anticoagulation: Group Citrate: Patients only received hemodialysis with citrate anticoagulation during the ICU stay; Group Heparin: hemodialysis with systemic heparin anticoagulation; Group Both: hemodialysis with citrate and heparin anticoagulation. The physicians in the ICU decided the anticoagulation regimens for SLED. All dialysis treatments were supervised by nephrologists.

Demographic and clinical data including laboratory and the Simplified Acute Physiology Score II (SAPS II) upon ICU admission were collected from a hospital information system.

Data regarding SLED monitoring, interruptions and the cause of interruption were evaluated from the routinely filled work sheets by dialysis nurses or nephrologists.

### SLED and citrate-based protocol

SLED, usually lasting 8-12 hours, was conducted using the GENIUS 90 therapy system (Fresenius Medical Care, Bad Homburg, Germany) and helixone filters (FX 60: 1.4 m^2^, 46 ml/h x mmHg; Fx 40: 0.6 m^2^, 20 ml/h x mmHg; Fx 80: 1.8 m^2^, 59 ml/h x mmHg).

For SLED with RCA, 30% trisodium citrate (SERACIT, SERAG-Wiessner GmbH, Bayer: citrate 1000 mmol/l) was infused into the arterial line, and calciumchloid-dihydrate (SERAG-Wiessner GmbH, Bayer: 0.5 mmol/ml) into the venous line. The dialysate calcium was 1.0 mmol/l. Judgment of citrate flow rate and calciumchloid-dihydrate flow rate according to the blood flow rate is shown in Table 1. The post-dialyzer iCa concentration was measured to assess individual situation, with a desired target range from 0.35 - 0.45 mmol/l.

**Table 1:** Citrate protocol for SLED according to filter types

Filter	System	Blood flow (ml/h)	Citrate (ml/h)	Caclium (ml/h)
FX 60 FX 80	1:1	140 - 160	60	10
	1:1	160 - 180	65	12
	1:1	180 - 200	70	14
	1:2 for 24h	140 - 160	45	8
FX 40	1:1	120 - 140	40	8
	1:1	140 -160	50	8
	1:2 for 24h	140 - 160	40	10

Blood gas analysis including Na^+^, K^+^, Ca^2+^, pH, bicarbonate for monitoring SLED with RCA was analyzed every 2h. Post filter ionized calcium levels were measured 30–60 minutes after initiation of SLED and rechecked if demanded.

For SLED with systemic heparin anticoagulation, heparin was given with or without bolus according to the value of activated partial thromboplastin time (aPTT) before dialysis and the physician’s discretion. The heparin infusion rate was adjusted to maintain an aPTT of 50–70 sec.

### Safety and efficacy

For safety, mortality of patients and adverse events related to SLED were studied. Meanwhile, we also looked into bleeding events and metabolic complications. Unexpected bleeding episodes associated with dialysis anticoagulation were considered as bleeding events. Patients were considered to be at high risk of bleeding for heparin anticoagulation if they were undergoing active bleeding, within the initial 24 h after invasive intervention (puncture, biopsy), or having acute decrease in hemoglobin (> 2 mg/dl within 24h).

Metabolic complications included incorrigible electrolyte imbalance, metabolic alkalosis or metabolic acidosis which led to interruption of SLED.

In addition, we compared the in-hospital mortality in patients with different anticoagulation regimens. The predictive factors of mortality were SAPS II score on ICU admission.

For efficacy, interruption rates of SLED, reasons of interruption, filter clotting and circuit survival times were investigated. Interruptions of SLED were usually due to unexpected extracorporeal circuit clotting, catheter malfunction, diagnostic procedure or other complications.

Furthermore, we analyzed treatment parameters at 0, 2, 4, 6, and 8 hours of SLED with citrate anticoagulation.

### Cost

We assessed hemodialysis nurse fee and the material cost for a 10 hour dialysis. This cost included costs of dialysate and fluid, anticoagulant, the cost of laboratory assignments, tubings, dialysis machine, and filter set. The capital cost of the dialysis machine, and the physician fees were excluded.

### Statistical analysis

Data are expressed as mean ± SD or median (Interquartile range: Q1-Q3) for continuous variables and as absolute and relative frequencies for categorical variables. Group comparisons for independent data were performed by chi square (X^2^) test, Kruskal Wallis test or Mann-Whitney U test, where appropriate. Association between SAPS II score and in-hospital mortality was assessed using a logistic regression model. When multiple observations were available for the same patient (repeated SLED sessions), generalized estimating equations (GEE) models were fit to the data to account for within-subject correlation.

To assess the change of treatment parameters related to regional citrate anticoagulation over time, we fit nested linear mixed models to the data to account for the correlation structure present in the data (different timepoints assessed within one dialysis, multiple dialyses of the same patients).

A *P<* 0.05 was considered to be statistically significant.

All statistical analyses were performed using IBM SPSS Statistics Version 22 and R-3.3.0.

## Results

### Patients Characteristics

A total of 349 critically ill patients received SLED during the observation phase (Table 1). Sixty-seven Patients underwent SLED with other anticoagulants, without anticoagulation, or longer than 12 hours were excluded. Two hundred eighty-two patients who were treated with regional citrate or systemic heparin anticoagulation were retrospectively analyzed. Seventy-five patients at high risk of bleeding according to assessments by physicians were treated with RCA (group Citrate), 79 with systemic heparin anticoagulation (group Heparin), 128 patients who had risks of bleeding during the ICU stay received both citrate and heparin anticoagulation (group Both). Sample sizes were not predetermined for any statistical advantage. Heterogeneity between groups was observed. In group Both, the anticoagulation was either changed from heparin to citrate or vice versa. Thirty-four patients have been changed from heparin to citrate due to bleeding, 6 patients on suspicion of HIT. The other patients received firstly citrate anticoagulation while they had a risk of bleeding such as operation, intervention, active bleeding.

The groups differed significantly in number of dialysis sessions per patient and time of hospitalization per patient (Table 2). However, those differences were not observed between group Citrate and group Heparin (*p=* 0.97, *p*= 0.85). Acute kidney failure was present in 227 (80%) patients. The overall in-hospital mortality was 49%. Severity of acute illness, as reflected by the SAPS II score on admission, was higher for patients in group Citrate and in group Both, as compared to group Heparin.

**Table 2:** Baseline characteristic of patients

Characteristic	All	Citrate	Heparin	Both	*p*
Patients	282	75 (27%)	79 (28%)	128 (45%)	
Age	68 ± 13	67 ± 12	69 ± 14	69 ± 14	0.25
Dialysis per patient^b^	6 ± 7	4 ± 4	4 ± 4	9 ± 9	< 0.001
Female	101 (36%)	28 (37%)	30 (38%)	43 (34%)	0.89
Race					
White	256 (91%)	68 (91%)	73 (92%)	115 (90%)	0.99
Asian	5 (2%)	3 (4%)	0	2 (2%)	
Arab	20 (7%)	4 (5%)	0	10 (7%)	
Black	1 (0,3%)	0	6 (8%)	1 (1%)	
Sepsis	122 (43%)	26 (35%)	34 (43%)	62 (48%)	0.36
Liver cirrhosis	18 (6%)	9 (12%)	3 (4%)	6 (5%)	0.07
ARF	227 (80%)	60 (80%)	71 (90%)	96 (75%)	0.49
Anuria/oliguria	153 (67%)	37 (62%)	45 (63%)	71 (74%)	0.51
MOF	19 (25%)	32 (33%)	24 (30%)	50 (39%)	0.54
Invasive mechanical ventilation	213 (76%)	51 (68%)	54 (68%)	108 (84%)	0.31
Needs of catecholamine	202 (72%)	50 (67%)	51 (65%)	101 (79%)	0.44
Time of hospitalization (day)^a^	18 (8-32)	14 (4-29)	14 (6-26)	22 (11-40)	< 0.001
SAPS II score	44 ± 14	45 ± 14	42 ± 13	44 ± 14	0.504
In-hospital mortality	138 (49%)	34 (45%)	34 (43%)	70 (55%)	0.42

Continuous variables are present as mean ± SD or median (Interquartile range: Q1-Q3)

^a^ Categorical variables are present as frequency (n) and percentage (%)

^b^ Significant difference among groups. No significant difference between group Citrate and group Heparin

### Safety

Two aspects of safety were considered: mortality of patients and complications of anticoagulation regimens.

To avoid misleading results caused by inadequate observation time, in-hospital mortality with an observation time ranged from 1 day to 221 days was investigated.

**Fig. 1:**
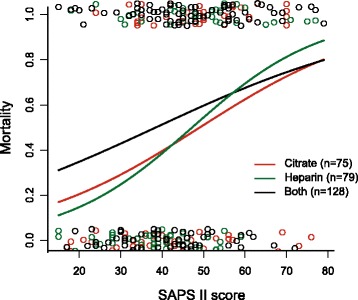
The in-hospital mortality among different anticoagulation regimens. Graphic illustrated the in-hospital mortality among different anticoagulation regimens. The predicted factor was the SAPS II score on ICU admission. Data was analyzed using binary logistic regression. Each dot on the scatterplot represents one patient

Figure 1 shows the in-hospital mortality of all patients. As expected, higher admission SAPS II Score was associated with increased mortality risk (*p<* 0.001). Mortality rates did not differ among groups (*p*= 0.42). After adjusting for age, days in hospital, SAPS II score, race, and sex, we could not observe a statistically significant difference among anticoagulation regimes (*p*= 0.33).

A total of 207 patients received SLED with heparin during their stay in the ICU: 79 patients only received SLED with heparin (group Heparin), 128 received both heparin anticoagulation and citrate anticoagulation for SLED (group Both). Hemorrhagic complications occurred in 37 of the 207 patients (18%): Three patients in group Heparin passed away due to unexpected acute bleeding: one patient with cerebral bleeding, one with intra-abdominal bleeding, and one with pulmonary bleeding. The anticoagulation in 34 patients in group Both has been change from heparin to citrate due to unexpected bleeding.

Metabolic complications which led to interruption of SLED occurred in 4 SLED sessions with RCA (0.5%: 2 increased metabolic acidosis, 1 derangement of sodium, 1 uncontrollable hyperpotassemia), and 1 session in SLED with heparin anticoagulation (uncontrollable hyperpotassemia). Hypotension occurred more frequently, though not statistically significant, in SLED with citrate anticoagulation (Table 3).

**Table 3:** Treatment parameters of SLED using citrate and systemic heparin anticoagulation

Parameters	SLED session with Heparin n (%)	SLED session with Citrate n (%)	*p*
Dialysis access	976	808	0.11
Fistula/Shunt	149 (15)	101 (13)	
Non-tunneled catheter	682 (70)	615 (76)	
Tunneled catheter	145 (15)	92 (11)	
Dialysis filter			
Fx40	55 (6)	40 (5)	0.76
Fx60	907 (93)	757 (94)	0.94
Others	14 (1)	11 (1)	
Extracorporeal circuit clotting	95 (10)	38 (5)	< 0.001
Interruption	167 (17)	84 (10)	< 0.001
Circuit clotting	95 (57)	38 (45)	< 0.001
Fistula/Shunt	7 (7)	3 (8)	0.76
dialysis catheter	88 (93)	35 (92)	
Catheter malfunction	33 (20)	19 (23)	0.67
Machine/shunt problems	7 (5)	3 (4)	0.76
Bleeding	3 (2)	0	
Metabolic complications^a^	1 (0)	4 (5)	0.06
Hypotension	2 (1)	3 (4)	0.23
Death/CPR	10 (6)	7 (8)	0.95
Diagnostic procedure or surgery	16 (10)	10 (12)	0.02

2 increased metabolic acidosis, 1 derangement of sodium, 1 uncontrollable hyperpotassemia

1 SLED session with heparin anticoagulation was broken due to uncontrollable hyperpotassemia

SLED. *P*-value was analyzed using generalized estimating equations (GEE)

*CPR* cardiopulmonary resuscitation: Patients received cardiopulmonary resuscitation during

^a^ 4 dialysis sessions performed with citrate were interrupted due to metabolic complication

### Efficacy

To measure the efficacy of the citrate-based protocol for SLED, we compared citrate with heparin anticoagulation in incidence of SLED interruption and average treatment duration as shown in Table 3.

Data concerning SLED efficacy were collected from all 3 groups. A total of 976 SLED sessions performed with heparin and 808 with citrate were collected from 282 patients.

In this study, PTT was controlled in 460 out of 976 SLED sessions with systemic heparin (47%). In detail, the PTT goals were achieved in 181 controlled sessions (39.4%). In the other controlled sessions, PTT was either below the range (144 sessions, 31.3%) or above the range (135, 29.3%).

In total, 167 (17%) of the 976 SLED sessions performed with heparin and 84 (10%) of the 808 sessions with citrate were interrupted.

Irreversible clotting of the extracorporeal circuit occurred more frequently in heparin anticoagulation (10%) than in RCA (5%) (*p*< 0.001).

As presented in detail in Table 4, ionized calcium concentration changed significantly during SLED in the direction towards normalization. All parameters changed over time. But most of the changes were minimal. The infusion dosage of citrate solution and CaCl_2_ according to the protocol, as mentioned in Materials and Methods, was able to maintain therapeutic circuit and ionized calcium concentration of patients. Neither hypocalcemia nor hypercalcemia was observed.

**Table 4:** Treatment parameters related to regional citrate anticoagulation. Data are present as mean ± SD

	Basal	2h	4h	6h	8h	*P*
Systemic iCa++ (mmol/l)	1.13 ± 0.11	1.11 ± 0.08	1.1 ± 0.07	1.1 ± 0.07	1.11 ± 0.07	<0.001
Systemic HCO3- (mmol/l)	21.8 ± 3.9	21.7 ±.3.3	22.1 ± 3.3	22.2 ± 3.3	22.2 ± 3.4	<0.001
Systemic PH	7.34 ± 0.1	7.33 ± 0.1	7.35 ± 0.1	7.35 ± 0.1	7.35 ± 0.1	<0.001
Systemic Na+ (mmol/)	133.7 ± 5.5	133.9 ± 5.0	134 ± 4.5	134 ± 4.1	134.1 ± 4.0	<0.001
Blood flow rate (ml/min)	150 ± 15	150 ± 15	150 ± 15.8	150 ± 15.4	150 ± 12.8	0.003
Citrate infusion rate (ml/h)	59.3 ± 6.5	59.9 ± 7.2	60.2 ± 7.2	60.4 ± 7.1	60.4 ± 6.7	<0.001
Calcium infusion rate (ml/h)	9.5 ± 2.3	9.5 ± 2.7	9.3 ± 2.9	9.3 ± 3.0	9.2 ± 3.0	<0.001
Postfilter iCa++ (mmol/l)	0.47 ± 0.1	0.46 ± 0.1	0.44 ± 0.1	0.42 ± 0.1	0.41 ± 0.1	<0.001
Filter citrate removal (%)		66	66	70	70	

Data was analyzed using binary logistic regression. Each dot on the scatterplot represents one patient. *P*-value was analyzed using nested linear mixed model

### Cost

Depending on the situation in each SLED, the total costs of hemodialysis nurse fee, dialysate and fluid, laboratory assignments, tubings, dialysis machine, and filter set fluctuate between 130€ and 195€. To be specific, the anticoagulant for a 10-h SLED cost 15€ for the 30% citrate solution, 5€ for heparin, and 55€ for the ACD-A solution.

## Discussion

To the best of our knowledge, this is the first reported citrate-based protocol for SLED using 30% citrate, and could be one of the largest studies regarding anticoagulation regimens for SLED. The citrate-based protocol for SLED using 30% citrate solution is safe, efficacious and cost-efficient.

### Safety

As reported previously, SAPS II score on administration correlated with patient’s outcome (*p* < 0.05), which has also been observed in our study.

Only a few studies estimated the vital outcome of critically ill patients who underwent citrate dialysis in comparison with heparin dialysis. Consistent with some previous studies, mortality of patients in our study did not significantly differ among anticoagulation regimens. Some trials observed beneficial effects of citrate CVVHD on patient’s mortality in comparison with low-molecular weight heparin, whereas others did not show any benefit for citrate [21–23].

Most randomized trials had small sample size and high patient selectivity. Patients at high risk of bleeding have to be excluded in randomized trials. However, a substantial portion of the patients in ICU is at high risk of bleeding. The exclusion of those patients may lead to an incorrect readout. In our study, a total of 72% of our patients (27% lasting, 45% temporary) were at risk of bleeding. Those patients should be considered for safety evaluation.

A critical complication of heparin anticoagulation is increased risk of bleeding. Bleeding events, depending on the dosage of heparin, were reported in 10–50% of cases [8, 9]. Consistent with previous studies, the incidence of bleeding for SLED with heparin was 18%. Three patients (2%) were died of heparin-induced bleeding.

We are not able to assess the incidence of bleeding in SLED with citrate. Some of the patients were already undergoing active bleeding before SLED. Thus, it is difficult to determine if the bleeding was caused by anticoagulation.

Another concern regarding the safety of this citrate protocol could be the high concentration of the citrate solution used in our study. Until now, the highest concentration of citrate solution used in citrate anticoagulation for dialysis was reported to be 15%. To note, a low concentration of citrate solution does not guarantee the safety of dialysis treatment. None of the citrate solutions reported in previous protocols can be delivered directly intravenous. A possible citrate accumulation could be excluded in none of those citrate-based protocols. The complications described in our study were also reported by other trials using low concentration citrate.

The main drawback of RCA is metabolic disturbances, which were also presented in our study [24–26]. At a rate of 0.5 percent, the metabolic complications of RCA including acidosis and alkalosis were higher, though not statistically significant, than that of heparin anticoagulation. Interestingly, one SLED session with heparin was also interrupted due to persistent hyperpotassemia. A possible explanation is that GENIUS hemodialysis system is a closed tank dialysis system with prepared dialysate which cannot be adjusted during the dialysis session.

An advantage of our protocol over the protocol with ACD-A solution could be a reduced volume overload. As mentioned above, a significant reduction of infused volume using 15% citrate protocol contrasting with using 4% citrate has been detected [19]. We assume that our citrate-based protocol could also provide similar beneficial effects on reduction of volume shifting.

### Efficiency

Data comparing the efficacy among different anticoagulation regimens for SLED is scanty.

Depending on the dialysis system a circuit clotting rate ranged from 0 to 15% for a 6–8 h SLED using citrate-based protocol was reported by several trials [10]. Separate data for heparin and citrate anticoagulation was not available.

In contrast to our results, the extracorporeal circuit clotting with Genius system has been reported to be less of a problem. In a trial of 20 patients who received heparin anticoagulation for SLED using Genius system, no extracorporeal circuit clotting was detected [27, 28]. However, the sample size of this study was small, and all patients received 1000 units of heparin initially and 500 units per hour afterwards. Not all patients could tolerate this dosage of heparin. The heparin bolus could be the trigger of a bleeding event. In a practical clinical setting, physicians usually minimize the dosage of heparin to reduce the risk of bleeding. In our study, the PTT goal was only achieved in 181 sessions (18.5%) of the SLED with heparin. This explained the observation of significantly higher incidence of circuit clotting in heparin anticoagulation (10%) in our study, as compared with citrate (5%, *p* < 0.001). The circuit clotting would increase the cost of treatment. Even more importantly, the extracorporeal clotting could risk patients’ health.

### Costs

Apart from the complications, an additional barrier that might impede the clinical utilization of RCA for SLED is the cost. Comparative data regarding the cost between different anticoagulation regimens for SLED is lacking. One of the most commonly used solutions for RCA is ACD-A [29]. However, not all hospital can afford the ACD-A solution.

The citrate protocol using 30% citrate solution was initially developed for CRRT using multiFiltrate (Fresenius medical care, Germany) instead of Ci-Ca system with 4% citrate solution, resulting in a reduction of 50% of the total dialysis cost. The SLED with RCA was at first performed with ACD-A. However, the clinical implementation was limited due to the high cost and complexity of the protocol. We thus modified a protocol with 30% for SLED. The cost of anticoagulant in our protocol is three times less than the protocol with ACD-A. The cost difference is not relevant for a single 10-h SLED, but becomes greater with increasing duration and frequency of SLED.

### Limitations

Several limitations of our study need to be addressed. First, it is not a randomized controlled trial. However, patients with bleeding risk such as post-operation, active bleeding, or post puncture could not be included in randomized trials of regional citrate versus heparin anticoagulation. The patients in randomized trials are not representative of the entire patient population in ICU.

Secondly, we were not able to collect enough data on patients with liver failure. These patients were usually not treated by nephrologists or received dialysis without anticoagulation. As reported, the citrate clearance is reduced in patients with liver failure, which could be associated with increased risk of metabolic complications. Therefore, in patients with liver failure, we recommend increased caution of application of citrate-based protocol, and more frequent measurements of electrolytes.

## Conclusion

In conclusion, the citrate-based protocol for SLED described in this study was safe, efficacious, and cost-effective. Additionally, this study provides several novel insights into the safety, efficacy, and cost of citrate anticoagulation SLED.

## Abbreviations

ACD-A: Acid citrate dextrose solution A
AKI: Acute kidney injury
aPTT: Activated partial thromboplastin time
CaCl_2_: Calcium chloride
CPR: Cardiopulmonary resuscitation
CRRT: Continuous renal replacement therapy
CVVHD: Continuous venovenous hemodialysis
ICU: Intensive care unit
PIRRT: Prolonged intermittent renal replacement therapy
RCA: Regional citrate anticoagulation
SD: Standard deviation
SLED: Sustained low-efficiency dialysis
